# Ovarian Cancer Staging—How CT Scan Descriptions Differ from Surgical Findings

**DOI:** 10.3390/jcm13154560

**Published:** 2024-08-05

**Authors:** Adrianna Ćwiertnia, Dominika Borzyszkowska, Anna Golara, Natalia Tuczyńska, Mateusz Kozłowski, Wojciech Poncyljusz, Agnieszka Sompolska-Rzechuła, Katarzyna Kotrych, Aneta Cymbaluk-Płoska

**Affiliations:** 1 Department of Reconstructive Surgery and Gynecological Oncology, University Clinical Hospital No. 2, Pomeranian Medical University in Szczecin, Al. Powstańców Wielkopolskich 72, 70-111 Szczecin, Poland; domi98nikab@gmail.com (D.B.); nataliat501@gmail.com (N.T.); aneta.cymbaluk@gmail.com (A.C.-P.); 2 Department of Diagnostic Imaging and Interventional Radiology, University Clinical Hospital No. 1, Pomeranian Medical University in Szczecin, 71-252 Szczecin, Poland; wojciech.poncyljusz@pum.edu.pl; 3 Department of Applied Mathematics in Economics, Faculty of Economics, West Pomerania University of Technology Szczecin, Janickiego 31, 71-270 Szczecin, Poland; agnieszka.sompolska-rzechula@zut.edu.pl; 4 Institute of X-ray Diagnostic and CT, USG and MRI Scanning Workroom, University Clinical Hospital No. 2, Pomeranian Medical University in Szczecin, 70-204 Szczecin, Poland; kotrych1@gmail.com

**Keywords:** ovarian cancer, cytoreduction, debulking surgery, computed tomography, oncology, gynecologic oncology

## Abstract

Ovarian cancer is one of the most common causes of cancer death in women worldwide. Most often, it is detected in an advanced stage due to its insidious onset and lack of symptoms in stages I and II. That is why imaging diagnostics is so important. Therefore, we assessed the consistency of the image seen on CT with the actual image assessed during surgery. **Objectives**: The aim of this study is to compare preoperative evaluation based on CT reports with those obtained during ovarian cancer surgery to determine whether CT is helpful in assessing the possibility of optimal or complete cytoreduction. **Methods**: This retrospective study included patients diagnosed with ovarian cancer who underwent diagnostic laparoscopy or laparotomy with cytoreduction. We compared ovarian cancer lesions described by radiologists on CT scans to those described during laparoscopy or laparotomy; the Wilcoxon signed-rank test for paired observations was used to compare the variables. **Results**: We observed that the morphology of the tumor, mesenteric infiltration, and the assessment of the involvement of the abdominal, para-aortic, and iliac lymph nodes may differ in CT examination and during surgery. **Conclusions**: The site of the tumor exit on a CT scan does not always reflect the original site seen during surgery.

## 1. Introduction

According to the 2020 Globocan report, ovarian cancer is the seventh most common cancer and eighth most common cause of death from cancer in women in the world. In Poland, there were 3012 occurrences of ovarian cancer among females in 2020, according to data from the National Cancer Registry (KRN), but there were 2688 fatalities from the disease. In 2020, the incidence rate of ovarian cancer was 8.8 per 100,000 women, while the mortality rate was 6.2 per 100,000, both standardized (with respect to the global population) [1]. Around 207,000 people worldwide lose their lives to ovarian cancer each year, and about 31,000 new cases are diagnosed. These figures are expected to rise over the next few years. It is likely that by 2040, there will be 445,721 cases of ovarian cancer worldwide and 313,617 deaths from the disease among women [2]. Ovarian cancer is most often detected at an advanced stage [3]. Insidious onset, lack of symptoms in grades I and II, or nonspecific symptoms such as abdominal pain, bloating, and diarrhea delay diagnosis [4]. There is currently no adequate test available that could be used as an ovarian cancer screening method [4,5]. In addition to the stage of ovarian cancer, appropriate treatment is equally important, which also affects prognosis. Five-year survival rates for women with ovarian cancer in more developed countries range between 36% and 46%. In contrast, there are countries where the rate is much lower. Such statistics indicate that access to and the use of proper treatment play an important role. A 5-year survival rate of 50% and a longer progression-free time (PFS) may be possible in those patients who achieve complete tumor cytoreduction [6] because it is the lack of residual disease that translates into longer patient survival compared to patients who have had optimal surgery leaving residual disease (<1 cm) [7]. According to some data, overall survival after 6 months after achieving optimal cytoreduction can be up to 100%, and after 12 months 92.8%. However, in patients with 0.1–1 cm of residual disease, survival is closer to 74.1% at both 6 and 12 months. For residual disease >1 cm, survival may be close to 50% at 6 and 12 months. An assessment of the chances of complete cytoreduction (CCR) should be made before surgery [6]. If CCR is unlikely, surgery is deferred in favor of neoadjuvant chemotherapy [8]. On the other hand, if tumor conditions permit primary debulking surgery, the overriding goal is to complete the operation without leaving macroscopic residual disease. It is residual disease that has a high prognostic value [9]. Despite the wide use of computed tomography in the staging of ovarian cancer, its predictive value, sensitivity, and specificity may vary depending on the anatomical region [10]. There are attempts to rely on indices that will enable contrast-enhanced computed tomography (CT) evaluation. The limitation of CT is primarily the inability to mark small extraintestinal deposits (<5 mm), i.e., on the serous membrane of the intestine, mesentery, and peritoneum. A major role in imaging with CT has been played by multiplanar reformatting, so that now subdiaphragmatic areas are no longer as challenging as they once were and can be imaged and evaluated [11]. Lesions that have a high probability of not being resectable with complete or optimal cytoreduction include implants >2 cm, diaphragm, lesser sac, porta hepatis, intersegmental fissure, gallbladder fossa, gastrosplenic and gastrohepatic ligaments, and small bowel mesentery, as well as lymph node enlargement above the renal hilum, abdominal wall invasion, and parenchymal and subhepatic liver metastases [12]. In addition to improving CT, work is underway to explore the usefulness of MRI in the staging of ovarian cancer, including the diffusion-weighted magnetic resonance imaging method (WB-DWI/MRI). WB-DWI/MRI may prove to be a better tool for assessing the primary tumor, staging, and predicting incomplete resection in patients with suspected ovarian cancer than CT [13].

Due to the fact that the actual picture of cancer observed during surgery may differ from the results of computed tomography, there is a need to systematize the radiological assessment in such a way as to make the right decision about subsequent treatment [11].

The purpose of our study was to point out the differences between CT and surgical descriptions of advanced ovarian cancer and to highlight anatomical areas that may be unclear on CT imaging, as well as during their interpretation by radiologists. This work makes it possible to point out which regions need special attention when deciding on further therapeutic steps, which in ovarian cancer depends on the chance of complete or optimal tumor cytoreduction. By highlighting the problems that still exist in professional communication between radiologist and surgeon, this work allows us to improve reproducibility and the links of CT with surgical results.

## 2. Materials and Methods

### 2.1. Assumptions and Objectives of the Study

**Figure 1:**
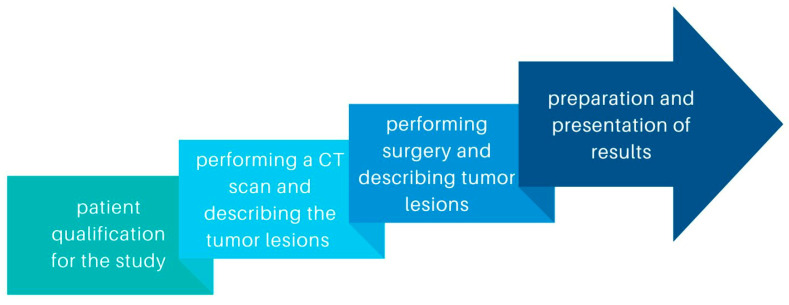
Diagram showing the stages of the study.

This retrospective study included patients diagnosed with ovarian cancer who underwent diagnostic laparoscopy or laparotomy with cytoreduction between 2019 and 2022 in University Clinical Hospital No. 1 of the Pomeranian University in Szczecin and University Clinical Hospital No. 2 of the Pomeranian University in Szczecin. We compared ovarian cancer lesions described by 6 radiologists on CT scans to those described during laparoscopy or laparotomy. The stages of the study are shown in the diagram (Figure 1). Measurable and non-measurable lesions were included. Measurable lesions have dimensions > or =10 mm on CT scan, with lymph nodes measuring > or =10 but <15 mm defined as non-measurable and > or =15 as measurable. Lymph nodes <10 mm are not pathological lesions. We also compared the concordance of such tumor features as tumor morphology and the involvement of specific organs or areas of the abdominal cavity: uterus, bladder, vasculature, peritoneum, mesentery, small intestine, large intestine, diaphragm, liver, spleen, and the presence of omental cake. Abdominal lymph nodes, periaortic lymph nodes, and lymph nodes of the iliac vascular region were included in the results. To obtain information on differences in tumor structure, solid and litho-cystic tumors were included. 

### 2.2. Participation in the Study

**Figure 2:**
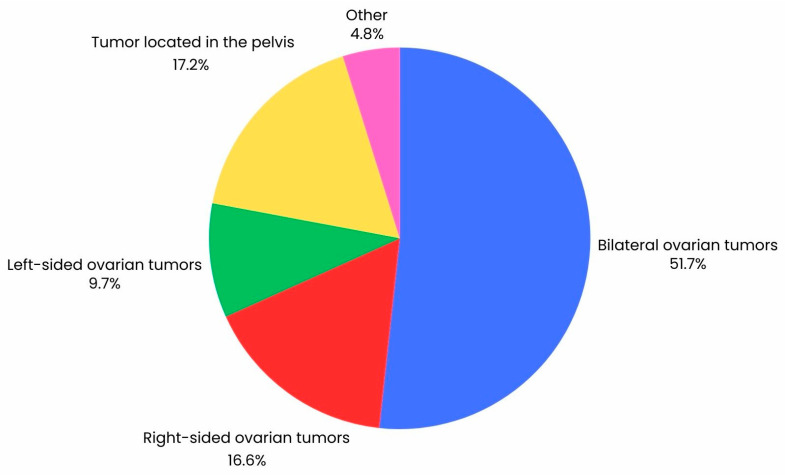
Graph illustrating the percentage distribution of the location of tumor lesions in a group of 147 ovarian cancer patients visualized by CT scan.

In this study, patients with histologically confirmed ovarian cancer were included in the analysis (n = 147*). The median age was 65 years (IQR = 14). In the retrospective study, features of ovarian cancer described on CT were compared with identical lesions described during laparoscopy (n = 65) and laparotomy (n = 109), including conversions of laparoscopy to laparotomy (n = 27). Exclusion criteria for the study included lack of patient consent, incomplete patient data, history of treatment for another cancer, pelvic inflammatory disease, and histological diagnosis of uterine malignancy other than cancer. All patients underwent a CT scan, followed by diagnostic laparoscopy, laparoscopy with conversion to laparotomy, or laparotomy. Figure 2 shows the percentage distribution of the location of tumor tissue visualized on CT. In addition, tumor dimensions obtained by CT scan and during surgery were compared using variables such as such as BMI, CA-125, HE4, ROMA index, recurrence, and cytoreduction. Each patient underwent contrast CT of the pelvis and abdominal cavity. Patients then underwent laparoscopy or laparotomies, and in some cases, laparoscopy was converted to laparotomy. Characteristics of the group are shown in Table 1.

### 2.3. Computed Tomography Examination

All CT examinations of the chest, abdomen, and pelvis to stage advanced OC were performed on a Siemens SOMATOM Edge Plus scanner with particular parameters: mA 35, 120 kVp, slice thickness: ≤0.625 mm, and interval: ≤0.5 mm with 4-phasic acquisition. SyngoVia (VB60A_HF07) radiological workstation was used to evaluate all CT scans. 

Negative oral contrast material (water) was used to visualize bowel loops and detect peritoneal deposits and mesenteric implants.

In patients, after evaluating renal parameters (glomerular filtration rate (GFR) and creatinine), obtaining consent, taking a history, and informing about possible complications after contrast administration, a CT scan was performed with Visipaque contrast solution for injection (652 iodixanolum mg/mL, 320 mg I/mL). The contrast dose was calculated based on the patient’s GFR. The patient was fasted during the study (6 h before the study without eating and 2 h without drinking). The patient was advised to drink increased amounts of water in the periods before and immediately after the study. None of our patients developed a complication after contrast administration.

### 2.4. Statistical Analysis

Because the samples studied in this paper were dependent, the Wilcoxon signed-rank test for paired observations was used to compare variables obtained from CT scan descriptions and after laparotomy or laparoscopy. This test belongs to the group of non-parametric tests [14]. The test ranks the results of differences between the measurements; that is, they are given weights in ascending order. In the test, a third variable is introduced to determine the absolute value of the difference between the values of pairs of observations. The calculated modules are assigned ranks. Separately, the ranks for negative differences and for positive differences are summed. The smaller of the resulting sums is the value of the Wilcoxon test statistic [15]. According to generally accepted statistical regulations, *p* value is α < 0.05.

In the Wilcoxon signed-rank test, when *p* > 0.05, there is no basis for rejecting the hypothesis that the tumor parameter on CT is equal to the tumor parameter from surgery, i.e., there are no significant differences between the tumor parameter on CT and after surgery. 

## 3. Results

### 3.1. Characteristics of the Group

The study included 147 women between the ages of 27 and 90. Detailed statistical characteristics of the patients’ age, body mass index, ovarian markers CA-125 and HE4, recurrence of cancer, and complete cytoreduction status are presented in Table 1.

### 3.2. Results of Wilcoxon Paired Rank Order Test on Comparing Preoperative Assessment Based on CT Descriptions to Those Obtained during Ovarian Cancer Surgery

In the Wilcoxon paired rank order test, if *p* > 0.05, there is no basis for rejecting the hypothesis that the tumor data described in CT scans are equal to the tumor data obtained during surgery. Then there are no significant differences between the tumor dimensions in the CT scan descriptions and the descriptions from surgery.

In this study, there were no statistical differences between tumor dimensions obtained by CT scan and those measured during surgery (*p* = 0.2750). Comparing the location of the tumor in the ovaries, a statistically significant result (*p* < 0.05) was obtained for the right ovary (*p* = 0.0328), which may mean that the described starting point of the tumor may differ between the CT scan result and the image obtained during surgery. For the left ovary, the results show that there is no basis for rejecting the hypothesis that there are differences between CT scan descriptions and surgical descriptions (*p* = 0.1227). A statistically significant difference was obtained for solid tumors (*p* = 0.0269), which means that the diagnosis of tumor structure differed between CT and surgical descriptions. However, for litho-tumor structure, the results show that there are no differences between the descriptions given (*p* = 0.2109).

In the comparison group of abdominal lymph nodes, abdominal, periaortic, and iliac vessel area lymph nodes were included. When comparing lymph nodes, statistically significant differences were obtained for abdominal, periaortic, and iliac vascular nodes, obtaining *p* = 0.00, *p* = 0.00, and *p* = 0.0433, respectively. A difference was also noted for uterine, vascular, and mesenteric lesions, obtaining *p* = 0.00, *p* = 0.00, and *p* = 0.00, respectively. Evaluation of the presence of pelvic fluid also showed differences between surgery and CT; *p* = 0.0020. In contrast, there were no statistically significant changes for peritoneum, small bowel, bladder, and omental, obtaining *p* = 0.1547, *p* = 0.1227, *p* = 0.838, and *p* = 0.5708, respectively. In the comparison of diaphragmatic and splenic lesions, the analysis showed no significant difference between CT and surgery (*p* = 1.0000 and *p* = 0.096, respectively). In contrast, a difference was noted in the liver lesions described (*p* = 0.0021) (Table 2).

Tumor dimensions were not statistically significantly different between CT descriptions and surgery in either of the two groups (for BMI > 25 *p* = 0.4929, for BMI < 25 *p* = 0.3097). No differences were also observed between the descriptions of the groups for which the median (Me) ROMA index > 90.6 (*p* = 0.2325) and in the other group ROMA < 90.6 (Me) (*p* = 0.4907). For the CA-125 marker, based on the two groups with CA-125 < 336.5 (Me) and CA-125 > 336.5 (Me), there were no statistically significant differences between the two descriptions in the group of patients with CA-125 < 336.5 (Me) (*p* = 0.666). In contrast, in the group of patients with CA-125 > 336.5 (Me), there was reason to reject the hypothesis that tumor dimensions were the same for CT descriptions and those performed during surgery (*p* = 0.0476). For differences according to the level of the HE4 marker, the results were as follows: for patients with HE4 > 286 (Me), *p* = 0.1064, and for patients with HE4 < 286 (Me), *p* = 0.4002 (Table 3). For patients without recurrence, *p* = 0.9288, and for patients with recurrence, *p* = 0.0367, which indicates that differences in tumor dimensions between CT descriptions and surgery are statistically significant for patients with tumor recurrence. For patients in whom optimal cytoreduction was achieved (size of remnants left <1 cm), *p* = 0.3220, and for patients in whom optimal cytoreduction could not be achieved, *p* = 0.4328. The results indicate that there are also no statistically significant differences between tumor dimensions described by CT and surgery in patients according to age. For the <65 years group, *p* = 0.4165, and for the >65 years group, *p* = 0.6893 (Table 4).

**Figure 3:**
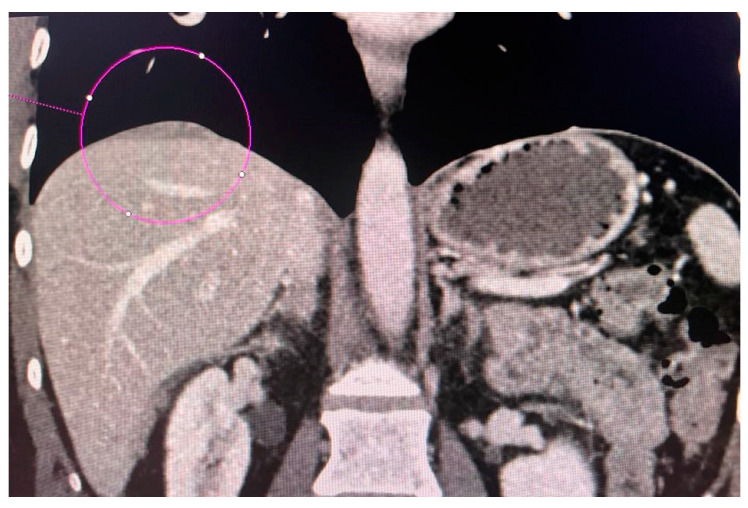
Conglomerate of implants near the diaphragmatic surface of the liver.

**Figure 4:**
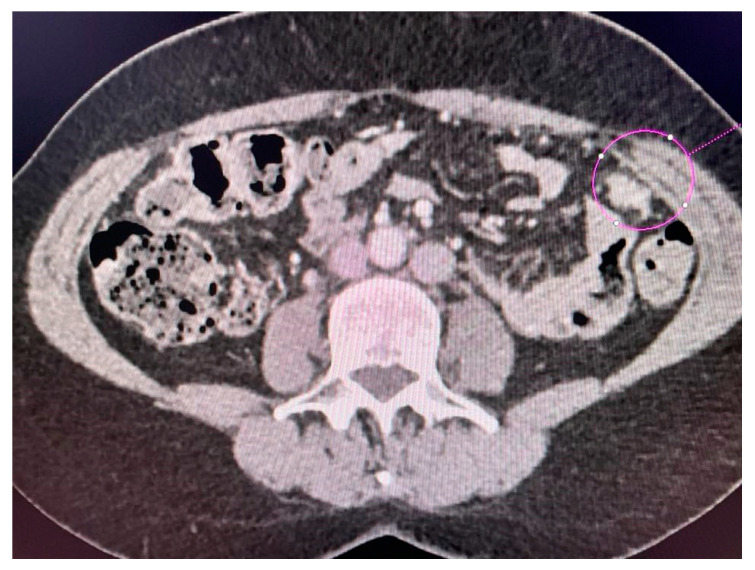
Left-sided tumor implant in the abdominal wall.

**Figure 5:**
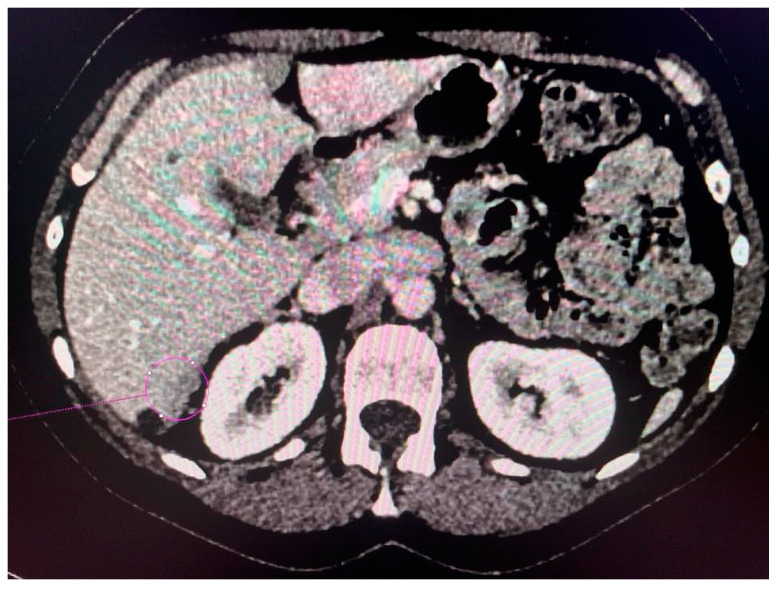
Tumor implants of the 6th and 7th liver segment.

CT scans obtained for a patient with advanced ovarian cancer are shown in Figure 3, Figure 4 and Figure 5. Patient age when the CT scan was taken was 51, and BMI was 29.8. The patient reported bloating and constipation. CA-125 at the time was 129 U/mL, and HE4 was 113 pmol/L.

Tumor diameter was 5.63 cm.

Scans were taken before chemotherapy.

### 3.3. Summary of Results

The results of this study show that differences between CT and surgical descriptions may be more noticeable for some anatomical areas, such as abdominal, periaortic, and iliac vascular node involvement (*p* < 0.05, *p* < 0.05, and *p* = 0.043, respectively) and liver metastases (*p* < 0.05). Determining the exact location of the tumor, let alone its starting point, is more difficult in CT, so the determinations of these two variables may sometimes differ from surgical descriptions (*p* = 0.06). Tumor structure was described differently for solid tumors (*p* = 0.03). Dimensions were not significantly different between the two descriptions (*p* = 0.28). In the comparison of diaphragmatic and splenic lesions, the analysis showed no significant difference between CT and surgery (*p* = 1.00 and *p* = 0.096, respectively).

## 4. Discussion

In the diagnosis of ovarian cancer, it is crucial to assess the peritoneum, searching for potential metastases as well as infiltrates in order to estimate the chances of performing surgery with achieving complete or at least optimal cytoreduction [16]. Evaluating peritoneal involvement on preoperative imaging studies is a difficult challenge. Still, when the evaluation is carried out correctly, it helps to detect unresectable regions and refer the patient for neoadjuvant chemotherapy [17].

Criteria for assessing surgical resectability are not commonly accepted, but each institution sets its own according to local surgical and oncological knowledge and the patient’s clinical condition. A systematic review of staging assessment using CT helps to identify resectable and unresectable sites of disease and to make decisions about the patient’s further therapeutic management [18]. Structured presentation of CT findings provides surgeons with important guidance on the course of surgery and allows for preoperative input from other surgeons whose assistance may be necessary to achieve complete resection of all visible disease (R0) or near complete resection, i.e., retaining a small volume of 0.1–1 cm (R1) of the disease. While resection is suspected to be impossible, CT plays a critically important role in identifying lesions > 2 cm of mesentery, gastrointestinal ligament, gallbladder, porta hepatis, falciform ligament, periappendiceal nodes, and lung parenchyma, as well as in detecting large retroperitoneal lymphadenopathy, extraperitoneal pre-sacral disease, and invasion of the lateral pelvic wall [16]. In contrast, a known limitation of CT in particular is its inability to determine small extraintestinal deposits (<5 mm), i.e., on the serous membrane of the intestine, mesentery, and peritoneum, even more so when the patient does not have ascites. Multiplanar reformatting has played a role in CT imaging, so now subdiaphragmatic areas can already be imaged and evaluated [11]. Our study showed that in some cases, radiologists are unable to assess the primary point of the tumor in CT scan descriptions, so the location described in the CT differs from that seen during surgery (*p* = 0.006). 

There are also studies evaluating the usefulness of spectral multiparameter computed tomography. It is proving to be valuable in the diagnosis of ovarian cancer. In addition, it is possible that spectral computed tomography can initially differentiate benign and malignant ovarian tumors [19]. In the study by Choi et al [20]., the sensitivity and specificity of CT for detecting peritoneal seeding were 45 and 72%, respectively. Moreover, in the same study, CT showed the highest sensitivity in the splenic hilum area (71%) and the lowest sensitivity in the bladder dome (0%) [20]. In our study, the splenic area assessed in CT scans was not strongly different from surgical descriptions (*p* = 0.096). In contrast to Choi et al.’s study, the assessment of the urinary bladder also did not show marked differences between CT scans and surgical descriptions (*p* = 0.838).

In 2006, Fagotti et al. created an objective quantitative model, based on laparoscopy, for predicting surgical outcomes using curve analysis to better assess the chance of optimal cytoreduction in patients with advanced ovarian cancer. The primary inclusion criteria for the study were the presence of net cancer, peritoneal cancer, diaphragmatic cancer, mesenteric retraction, intestinal and/or gastric infiltration, and liver metastasis. The final predictive index value assigned to these was two. In the final model, a predictive index score > or =8 meant patients would be undergoing suboptimal surgery, and the specificity was 100%. The positive predictive value was 100%, and the negative predictive value was 70%. Thus, this study showed that the assessment of optimal cytoreduction in patients with advanced ovarian cancer can be improved using a scoring system [21].

The Fagotti scale allows for assessing the possibility of optimal cytoreduction during surgery. The main advantage of a method such as the Fagotti scale is its reproducibility [22]. Fleming et al. conducted a radiological review of 20 patients with advanced-stage ovarian cancer who underwent laparoscopic evaluation. Their study is one of the first to evaluate the correlation of CT findings with laparoscopic findings by subscale of disease localization based on the Fagotti algorithm. The study showed that radiologic evaluation may not correlate well with actual laparoscopic evaluation results in patients with advanced ovarian cancer, regardless of the physician’s level of experience. The best concordance was observed for the evaluation of gastric involvement and the worst for liver involvement. The results suggest that the validated laparoscopic evaluation algorithm should be considered the gold standard for assessing the feasibility of primary resection in new cases of advanced ovarian cancer [23]. The study by Kim et al. analyzed the prediction of cytoreductive surgery outcomes in patients with advanced ovarian cancer based on CT scans and the Fagotti scoring system. Seven laparoscopic parameters were assessed intraoperatively: peritoneal carcinomatosis, diaphragmatic carcinomatosis, mesenteric retraction, omental cake, bowel and/or stomach infiltration, and superficial liver metastasis. Even between significant findings (such as peritoneal involvement, diaphragmatic carcinomatosis, mesenteric retraction, and omental cake), which are important diagnostic findings in laparoscopic diagnosis, there was no significant correlation [24]. In our study, the best concordance was observed for the evaluation of the bladder, peritoneum, and diaphragm and in the diagnosis of litho-tumor structure; the worst was for mesenteric lesions, vascular lesions, uterine lesions, liver lesions, and abdominal and periaortic nodes.

Than Singh Tomar et al. also used the Predictive Value Index (PIV) created by Fagotii et al. Inoperability was predicted in 12 of 14 inoperable patients using PIV, which was derived from laparoscopic data, preventing 85% of failed procedures [25]. The usefulness of the laparoscopic index of Fagotti et al. was also evaluated on four important parameters, such as mesenteric retraction, bowel infiltration, stomach infiltration, and liver metastasis during laparoscopy, when optimal cytoreduction should be predicted [26]. There have also been attempts to use a simplified index based on laparoscopy. In the study by Jean-Luc Brun et al., seven parameters were assessed, and a modified score was then determined by selecting four of the seven parameters. These were diaphragmatic cancer, mesenteric retraction, gastric infiltration, and liver metastasis. This method was shown to be at least as accurate as the Fagotti score to predict resectability [27]. The predictive ability of the CT-based model may become better with the integration of Eastern Cooperative Oncology Group (ECOG-PS) data. Adding ECOG-PS data led to improved diagnostic performance (z = 2.41, *p* value < 0.05) [28]. Laparoscopy reduces unnecessary laparotomies, which can be associated with a higher risk of complications for the patient [29].

S. Bendifallah et al. developed recommendations for cytoreductive evaluation considering, among other things, the importance of tumor markers, outcomes, and algorithms for diagnostic and prognostic purposes in the context of ovarian tumors suspected of being ovarian cancer [22]. Serum Cancer Antigen 125 (CA-125) and Human Epididymis Protein 4 (HE4) are recommended for the diagnosis of tumors suspected of malignancy on imaging studies. Of the selected markers, the above have the highest sensitivity and specificity [30]. However, it should be noted that the specificity of the CA-125 marker is lowered by an increase in its concentration also in noncancerous and inflammatory ovarian diseases [31]. Nevertheless, a CA-125 value of more than 500 IU/mL may signal a risk of suboptimal surgery. To assess ovarian tumor malignancy, the ROMA index is recommended. The ROMA index is calculated from equations that take into account CA-125 and HE4 levels, and these values are substituted into each equation depending on the patient’s age—premenopausal or postmenopausal. The ROMA index is better at predicting tumor malignancy than isolated serum CA-125 and HE4 marker values [32].

In our study, we assessed the effect of CA-125 levels on the evaluation of ovarian cancer resectability. We divided patients based on the level of this marker. The first group was patients with CA-125 < 336.5, and the second group was patients with levels of this marker > 336.5. For patients with CA-125 marker levels below (<336.5), there were no statistically significant differences in tumor dimensions between descriptions (*p* = 0.666). However, in the group of patients with concentrations >336.5, there may be grounds to reject the hypothesis that tumor dimensions are the same for CT descriptions and those performed during surgery (*p* = 0.048).

C. Herus et al. in their study pointed out that the visceral fat area (VFA) index is a better predictor of postoperative complications than BMI, as VFA more accurately correlates with the presence of abdominal obesity in a patient than BMI [33]. In our study, we intended to test whether there were significant differences in tumor size measured on CT and after surgery in the group of patients with BMI >25 and BMI < 25. We also compared tumor dimensions in patients divided into two groups: patients with BMI <25 and patients with BMI >25. Statistical analysis for both of the above groups showed that there was no basis for rejecting the hypothesis that tumor dimension on CT is equal to tumor dimension from surgery. That is, no significant differences were obtained between tumor dimensions on CT and during surgery (for BMI > 25 *p* = 0.4929, for BMI < 25 *p* = 0.3097).

Our study continued through the period of the global COVID-19 pandemic, which may have contributed to later diagnoses and therefore the diagnosis of ovarian cancers at higher stages. In addition to delaying medical appointments, the COVID-19 pandemic also forced hospitals to postpone several scheduled surgeries, including many cancer surgeries, in order to secure resources for the expected increase in COVID-19 patients [34]. In a study made in the United Kingdom, about 54% of women with ovarian cancer reported that their treatment was affected by COVID-19—some of the reasons included testing, active infection, and delays due to hospital inefficiency [31]. 

The above study suggested that in such situations, in patients with suspected ovarian cancer at an advanced stage of the disease, tissue biopsy should be considered to verify the diagnosis of the disease, neoadjuvant chemotherapy should be continued until the crisis subsides, and surgery should be considered at a later time [35]. In 2024, this matter is not an issue anymore, but we should consider similar diagnostic options so that if the pandemic returns, medical personnel and the healthcare system will be able to respond early and effectively in the diagnosis and treatment of ovarian cancer.

This study also has its limitations. One of the limitations of this study was the number of patients. The group consisted of 147 patients, but when comparing specific parameters, the number decreased. This was due to the heterogeneity of the group, as these patients had metastases and infiltrates detected at different sites. Seven radiologists described the CT results. Since this was a retrospective investigation, not a single radiology specialist recommended that the descriptions be utilized in future studies. The divergent perspective may have been impacted by professional experience. At the time that the descriptions were prepared, all of the radiologists who were describing the computed tomography data were already specialists; some had been so for ten years, while others had only been so for a year.

## 5. Conclusions

The results of this study show that when comparing most lesions, there is no basis for rejecting the hypothesis that the characteristics of the tumor, implants, infiltration, or metastasis are the same on CT as during surgery. Attention is paid to tumor morphology and mesenteric infiltration, the descriptions of which may differ. Among the lesions checked, the difference may also be important when assessing abdominal, periaortic, and iliac lymph node involvement. When evaluating the staging of ovarian cancer, differences are also evident in determining the exact location of the tumor. Based on this study, it can be concluded that the tumor exit site on CT does not always reflect the primary site seen during surgery. Further studies are needed to refine the preoperative assessment of the chances of cytoreduction in ovarian cancer. This will enable the appropriate classification of patients into groups and treatment and would help in the search for more accurate scores to identify patients with advanced ovarian cancer for optimal cytoreduction.

## Figures and Tables

**Table 1:** Characteristics of patients with ovarian cancer.

Characteristics	Number of Patients *
BMI	
<25	52
>25	58
Age [years]	
≤65	73
>65	69
CA-125 [U/mL] **	
>336.5	66
<336.5	66
HE4 [pmol/L] **	
>286	66
<286	65
Recurrence	
present	22
absent	60
Cytoreduction	
non-optimal ***	23
optimal ****	21

* In the results, the number of patients was lower because its value depended on the presence of a particular variable (such as metastasis to lymph nodes, liver, and peritoneum). Because of differences between the location of tumors in the patients presented, the final number of comparisons differed from the total number of participants. ** denotes concentrations determined before chemotherapy. *** denotes remnants left >1 cm. **** denotes remnants left <1 cm. BMI—body mass index, CA-125—Cancer Antigen 125, HE4—Human Epididymis Protein 4.

**Table 2:** Comparison of CT description and surgical description in the Wilcoxon test.

Locations of Tumor or Infiltration	*p* Value *
Right ovary	0.0328
Left ovary	0.1227
Solid tumor	0.0269
Litho-tumor structure	0.2109
Abdominal lymph nodes	<0.005
Periaortic lymph nodes	<0.005
Iliac vascular nodes	0.0433
Uterine lesions	<0.005
Vascular lesions	<0.005
Mesenteric lesions	<0.005
Pelvic fluid	0.0021
Peritoneum	0.1547
Small bowel	0.1227
Bladder	0.838
Omental	0.5708
Diaphragmatic	1.0000
Splenic lesions	0.096
Liver lesions	0.0021

* denotes the probability value; when *p* > 0.05, there is no basis for rejecting the hypothesis that the tumor parameter on CT is equal to the tumor parameter from surgery.

**Table 3:** Variation in tumor dimensions between CT descriptions and surgery in different groups.

Variable	Concentrations	*p* Value **
ROMA	>90.6	0.2326
	<90.6	0.4908
CA-125 [U/mL] *	>336.5	0.0476
	<336.5	0.6658
HE4 [U/mL] *	>286	0.1064
	<286	0.4003

* denotes concentrations determined before chemotherapy. ** probability value; when *p* > 0.05, there is no basis for rejecting the hypothesis that the tumor parameter on CT is equal to the tumor parameter from surgery. CA-125—Cancer Antigen 125, HE4—Human Epididymis Protein 4.

**Table 4:** Variation in tumor dimensions between CT descriptions and surgery in different groups.

Variable		*p* Value *
Recurrence	present	0.0367
	absent	0.9288
Cytoreduction	non-optimal	0.4328
	optimal	0.3220
Age	<65	0.4165
	>65	0.6893

* probability value; when *p* > 0.05, there is no basis for rejecting the hypothesis that the tumor parameter on CT is equal to the tumor parameter from surgery.

## Data Availability

The data presented in the study are available from the corresponding authors, M.K. and A.Ć., upon reasonable request.
